# Feline immunodeficiency virus latency

**DOI:** 10.1186/1742-4690-10-69

**Published:** 2013-07-06

**Authors:** Samantha J McDonnel, Ellen E Sparger, Brian G Murphy

**Affiliations:** 1 Department of Pathology, Microbiology & Immunology, School of Veterinary Medicine, University of California, Davis, 4206 Vet Med 3A, Davis, CA 95616, USA; 2 Department of Medicine and Epidemiology, School of Veterinary Medicine, University of California, 3115 Tupper Hall, Davis, CA 95616, USA; 3 VetMed PMI, 4206 VetMed 3A, One Shields Avenue, Davis, CA 95616-5270, USA

**Keywords:** FIV, Latency, HIV-1, Feline, Animal model, Antilatency therapy, Reservoir

## Abstract

Despite highly effective anti-retroviral therapy, HIV is thought to persist in patients within long-lived cellular reservoirs in the form of a transcriptionally inactive (latent) integrated provirus. Lentiviral latency has therefore come to the forefront of the discussion on the possibility of a cure for HIV infection in humans. Animal models of lentiviral latency provide an essential tool to study mechanisms of latency and therapeutic manipulation. Of the three animal models that have been described, the feline immunodeficiency virus (FIV)-infected cat is the most recent and least characterized. However, several aspects of this model make it attractive for latency research, and it may be complementary to other model systems. This article reviews what is known about FIV latency and chronic FIV infection and how it compares with that of other lentiviruses. It thereby offers a framework for the usefulness of this model in future research aimed at lentiviral eradication.

## Review

### Introduction

It was estimated that 34 million people worldwide were living with HIV/AIDS as of 2011, more than 1 million of whom were living in the United States [1]. While advancements made in highly active antiretroviral therapy (HAART) have dramatically increased survival time and quality of life for those infected with HIV, long term treatment is problematic for several reasons [2]. Among them are the necessity of life-long adherence to medication regimens, the potential for cumulative side-effects, emergence of drug-resistant mutants, and the unbearable cost for the majority of the world’s HIV-infected individuals [3,4]. In people undergoing HAART, viremia is typically reduced to less than 50 copies of HIV RNA per milliliter of blood [5]. Unfortunately, drug withdrawal generally results in rebound viremia, with subsequent progression to clinical AIDS [6]. It has been hypothesized that HIV is able to persist through both ongoing, low-level replication and as a transcriptionally inactive (latent) integrated provirus [7]. Studies demonstrating a lack of viral genetic evolution support the latter hypothesis, suggesting that cellular latency may be the cause of viral rebound [8], and memory CD4+ T cells are thought to be the primary long-lived reservoirs [9].

The concept of lentiviral latency has therefore come to the forefront of the discussion on the possibility of a cure for HIV infection in humans. For the purposes of this discussion, lentiviral cellular latency is defined as the presence of proviral DNA (integrated or episomal) in the absence of detectable viral RNA (vRNA) transcripts. Of course, such a definition is only meaningful in the context of highly sensitive real-time PCR assays as the inability to detect vRNA is not necessarily the same as a truly negative result. Cellular latency is distinct from clinical latency in that it describes the viral activity or inactivity within a single cell, rather than the collective manifestation of viral replication in the host as a whole. Latently-infected cells have been found to naturally occur in all three immunodeficiency-causing lentivirus infections [HIV, simian immunodeficiency virus (SIV), and feline immunodeficiency virus (FIV)] within their respective hosts [7,10-12]. Because latently-infected cells do not contain detectable viral RNA or protein, they go largely undetected by the host’s immune system. In addition, latent provirus is not affected by antiretroviral therapy (ART), which serves to impede only ongoing rounds of viral replication by inhibiting various viral enzymes or cellular entry mechanisms. Thus, the latently-infected cell population serves as reservoir for the persistence of HIV despite the presence of ongoing ART and represents the major barrier to viral eradication (cure) from the host [11,13,14].

Multiple molecular mechanisms may underlie the establishment and maintenance of latent cellular reservoirs including availability of transcription and nuclear export factors, the viral integration locus, RNA interference (RNAi), transcriptional interference, and epigenetic modifications of proviral DNA or histone proteins in the local chromatin environment [15-22]. It is thought that latently-infected cells, which are established within the first several days of infection, are stochastically (randomly) reactivated to resume viral transcription, translation, and virion assembly [20]. In the ART-treated individual, the likelihood that virions produced from transcriptionally reactivated cells will infect new cells is very low due to the constant presence of ART drugs in circulation. But in most cases upon removal of ART from an HIV-infected individual, viremia rapidly rebounds and causes an eventual progression to AIDS [11,14]. Thus understanding of how viral latency is established and maintained, and perhaps even more importantly, how it can be manipulated, is of great importance to viral eradication efforts. And if therapy to reverse viral latency (so-called anti-latency therapy, or ALT) is possible, it would be prudent to test this therapy in an animal model of lentiviral latency due to both apparent and inapparent risks involved with viral reactivation in an individual who is well controlled on ART. Therefore, animal models of lentiviral latency provide an essential tool to study mechanisms of latency and therapeutic manipulation. Three *in vivo* animal models of lentiviral latency exist today: the SIV-infected macaque, the HIV-infected humanized mouse, and most recently, the FIV-infected cat. This review seeks to describe what is known about FIV latency and how it compares with that of other lentiviruses, as well as provide a framework for the usefulness of this model in research aimed at lentiviral eradication.

### Current models of HIV latency

Each model of lentiviral latency has both distinct benefits and drawbacks [23]. The plethora of *in vitro* cell-line models of CD4+ T-cell latency have been instrumental in both mechanistic studies and in the screening of new classes of viral eradication drugs [24]. However, it is unclear how similar these proliferating cells are to the primary *in vivo* reservoir of resting/memory cells. In addition, since these models are often established by a single latently infected cell (*i.e.*, a clonal population), they may not reflect the heterogeneity of the latent reservoir in the host. Primary cell models have also been established which may more closely reflect HIV latency *in vivo*[25], but they are limited by their somewhat contrived induction. Latently-infected cells isolated from HIV-infected individuals and analyzed *ex vivo* may be the most reliable of the “*in vitro*” models, but they are more difficult to obtain in sufficient numbers (often requiring leukapheresis) and are still assessed in isolation from the immune system and *in vivo* environment. Animal models of lentiviral latency have therefore garnered much interest for investigations into the location and nature of viral reservoirs and potential induction therapy.

Humanized mouse models of HIV infection, based on engraftment of human cells and tissue into recipient immunocompromised mice, are undoubtedly the most tractable and versatile *in vivo* model. All of the benefits of using mice (cost, genetic traceability, and availability of reagents, among others), in addition to the fact that this model employs HIV-1 rather than another lentivirus, make it an attractive model for latency [26]. On the other hand, accurately modeling an infection that is so intricately related to the intact immune system is difficult in an incomplete or immunocompromised background. In addition, the inbred nature and non-natural host aspects of mouse models may be considered a disadvantage. The SIV-infected macaque, on the other hand, represents an outbred, large-animal model with a natural physiology close to that of humans. Using infected macaques treated with HAART regimens, SIV latency has been observed in peripheral blood, the central nervous system [27], and various lymphoid tissues [12], making this a strong model to study viral reservoirs that persist during therapy. However, nonhuman primate studies are expensive and time-consuming, and while HIV remains latent in humans for several years, this phase is abbreviated to several months in macaques [23]. The macaque monkey is also not a natural host of SIV. Like SIV, FIV represents an outbred, large animal model which is still experimentally tractable. In contrast, while SIV has greater genetic similarity to HIV, FIV infection in cats is the only case (other than HIV) of an immunodeficiency-causing lentiviral infection in its natural host. And unlike macaques, transgenic cats are becoming available for lentivirus-related research [28]. The cost and difficulty of using cats in research is much less compared to nonhuman primates. Given the complexity of the problem and the various strengths and weaknesses of each model, it may be concluded that each of these models, both *in vitro* and *in vivo*, have a role to play in the study of lentiviral latency, reservoirs, and eradication strategies.

### The FIV model of HIV infection

FIV was first isolated and described in 1986 from domestic cats with immunodeficiency-like syndromes in a northern California cattery [29]. The five major subtypes (clades) of FIV are designated A through E, and each has a particular geographic distribution throughout the world [30]. FIV is similar to HIV in genome structure and immunopathogenesis [31,32], and has been utilized as the only naturally-occurring animal model of immunodeficiency for HIV-infection in people [33]. Acute FIV infection results in flu-like symptoms including peripheral lymphadenopathy, neutropenia, and pyrexia [34-36]. During terminal stages of infection, animals exhibit feline acquired immunodeficiency syndrome (FAIDS), which includes opportunistic infections, lymphomas, wasting, and death [37]. As in HIV infection, there is typically a protracted asymptomatic phase lasting at least several years prior to the terminal immunodeficiency syndrome. Despite a lack of clinical signs, there is evidence of immunological impairment (CD4+ T cell depletion and CD4/CD8 ratio inversion) during the asymptomatic phase [38,39].

The genomes of FIV and HIV-1 (the predominant subtype of HIV) encode the same three main genes found in all retroviruses: ‘group-specific antigen’ (*gag*), polymerase (*pol*), and envelope (*env*), in addition to the lentivirus-specific accessory genes ‘viral infectivity factor’ (*vif*) and ‘regulator of virion expression’ (*rev*). HIV-1 encodes four additional accessory proteins not found in FIV: *tat*, *vpr*, *vpu*, and *nef*. FIV does however encode a unique accessory gene known as *orf-A*, thought to have functional overlap with *vpr*, *vpu*, and *nef*[40,41]. Importantly, the *orf-A* protein has not been shown to have significant transcriptional transactivating activity like HIV *tat*[42], and FIV is not known to encode any such transcriptional transactivator. In addition to genomic differences, the cellular tropism of FIV is generally thought to be broader than that of HIV as it includes all major subsets of mononuclear leukocytes [34]. However, both FIV and HIV have been shown to infect CD8+ T cells and B cells in addition to the CD4+ T cells and monocytes/macrophages, which are the primary permissive cell types *in vivo*[43-49]. Both viruses have also been reported to infect microglia, astrocytes, and various other cell types to a lesser extent [34]. The primary cellular receptors used by these lentiviruses are CD4 and CD134 for HIV-1 and FIV respectively [50-53]. Both viruses may use the chemokine receptor CXCR4, and HIV-1 can additionally use CCR5 as a co-receptor [54-57].

Despite intensive study since its discovery over 27 years ago, relatively little has been published on FIV in the chronic/asymptomatic, or even terminal FAIDS stages of disease. The vast majority of experimental FIV research has focused on acute FIV infection, with most studies terminating at or before 6 months post infection. This is in large part due to initial enthusiasm for FIV infection as a model for vaccine development and early immunopathogenesis [33,58], as well as the cost associated with long-term studies. As a result, relatively little is known about virological parameters during chronic FIV infection under experimentally controlled conditions. In naturally-infected cats, plasma viral RNA load has been shown to correlate with the clinical stage, survival time, and disease progression [59]. Similar to acute HIV infection, experimental FIV infection causes an initial undulating viremia lasting four to six months [10,31,34]. Diehl *et al*. described a decrease in plasma viremia after approximately 10 weeks of FIV-B infection, though it remained significant and detectable (~10^5^ copies/mL) until the end of the study period (36 weeks) [60]. The same group developed a model of rapid FAIDS progression by acute-phase FIV-C passage [61], and demonstrated with this accelerated model of pathogenesis that plasma viremia is predictive of FIV disease progression [62]. Miller and Fogle reported detectable viremia at 1, 2, and 3 years post infection with FIV-A [63]. Another study by Miller *et al.* found cell-free virus in cerebrospinal fluid and neural tissue at 350 days post intravenous infection with FIV-A, C, and an A/C chimeric virus [64]. Freer *et al.* reported stable, moderate plasma viremia and PBMC proviral burden after 1 to 7 years of experimental infection with FIV-B [65]. Kraase *et al.* found variably detectable proviral burden in cats infected with FIV-A for 322 weeks (~6 years), but significantly increased viral *env* evolution relative to 12 weeks post infection [66], suggestive of ongoing viral replication. In two studies of FIV superinfection, viral loads in both plasma and PBMC remained detectable over 9 months [67] or three years [68] in cats infected with just one subtype, but declined significantly or was undetectable in cats pre-infected with another, attenuated or chimeric subtype. Kohmoto *et al.* observed 3 experimentally FIV-infected cats over the course of 8 years, and found that the one animal that developed FAIDS had a very high plasma viral load (2^10^ titration) whereas the other two were undetectable [37]. Our research group has observed persistently undetectable plasma viremia using a sensitive real-time PCR assay after approximately 10 months of infection with FIV-C [10]. Other studies documenting the development of clinical signs and pathologic lesions after years of experimental FIV infection [69-74] have not examined plasma viremia or the status of intracellular virus replication. To summarize, plasma viremia and cellular proviral load during the chronic, asymptomatic phase of experimental FIV infection has been found to be quite variable, ranging from undetectable to 10^5^ copies/mL or higher, which may depend on viral subtype, inoculating titer, route of inoculation, or other factors.

More attention has been paid to immunological effects of long-term FIV infection, with a particular emphasis on the hallmark CD4+ T-cell depletion and persistent CD4/CD8 ratio inversion [38,39,75,76]. There has also been documentation of chronic immune dysregulation [75,77,78] and inadequate CD8+ T-cell antiviral function [79] in longitudinal studies. The immunophenotype of cells harboring latent FIV, which is largely uncharacterized for this virus, may affect the ability to pharmacologically reactivate latent virus. FIV has been shown to preferentially infect CD4 + CD25+ activated/regulatory T-cells (Tregs) [80], which correlates with both surface CXCR4 expression and binding of cellular transcription factors to the FIV promoter [81]. Feline Tregs have since been characterized using FoxP3 [82,83], so these studies are merely suggestive of infection in that subset. Importantly, the FIV receptor CD134 (OX40) is constitutively expressed on Tregs [84], lending support to this hypothesis. CD4 + CD25+ and CD4 + CD25- T cells appear to possess different activation requirements, modulated by viral titer and cytokine stimuli, to reach threshold activation levels required to harbor a productive FIV infection [85]. This holds implications for the differential ability of the two subsets to serve as potential latent reservoirs, though our research group has found both subsets to be equal in terms of latency status in the periphery during chronic FIV-C infection [10]. Finally, the ability of the immune system to adequately respond and kill reactivated cells is critical to proposed strategies to purge viral reservoirs. Selective depletion of CD4 + CD25+ cells has been shown to result in improved antiviral responses in cats chronically infected with FIV [83,86], which could potentially be part of a strategy to boost the immune system during or after ALT. There is much still to be learned about these and other interactions between the immune system and latent FIV.

### FIV Latency

Though the research has thus far been somewhat limited, several groups have observed FIV latency both *in vitro* and *in vivo*. Ikeda *et al.* demonstrated that an infectious molecular clone of FIV-Petaluma (FIV-A) was able to infect the human lymphoblastoid cell line MOLT-4 *in vitro*, but established a transcriptionally latent infection unless stimulated by phorbol ester [87]. The molecular mechanism of FIV latency in human cell lines has not been reported; however, this form of latency may be due to differences in species-specific viral restriction factors and corresponding viral evasion mechanisms. More recently, another group demonstrated that a cellular clone of a feline T-cell line (FeT-J) chronically infected (>50 days) with FIV-A led to a latent phenotype, which was inducible by treatment with mitogens [88]. There is also evidence that FIV can establish a latent infection *in vivo* following mucosal administration of low-dose cell-associated FIV-A [89], in peripheral blood CD4+ T-cells during chronic FIV-C infection [10], and in peripheral blood mononuclear cells (PBMC) during chronic FIV-B infection [90]. The later reported the presence of multiply-spliced FIV mRNA, but extremely low or undetectable levels of unspliced or singly-spliced mRNA in PBMC from FIV-B infected cats. The larger mRNA species, and production of infectious virus, could be rapidly induced by mitogen treatment. This is in contrast to our findings for FIV-C, in which we observed only short, promoter-proximal transcripts [91], similar to what was reported for HIV latency *in vivo*[92]. We have quantified the latent reservoir in peripheral CD4+ T-cells during asymptomatic phase of FIV-C infection to be approximately one in 10^5^ cells (1 in 10^3^ cells is infected, but only 1:100 of those is replication competent), with just one provirus per infected cell [91]. This figure is similar to that of HIV-infected humans in the asymptomatic phase [93,94]. Lastly, Uckun *et al*. report outgrowth of infectious virus from PBMC of cats chronically infected (> 6 months) with FIV-A, B, and D upon co-culture with specific pathogen free (SPF) T-cell-enriched PBMC [95]. While this is suggestive of latency, measures of viremia or cell-associated vRNA were not reported. The specific memory phenotype of CD4+ T cells that serve as a reservoir for FIV is currently unknown, but this question is actively being pursued.

A number of studies have used *in vitro* and *ex vivo* models to study mechanisms of FIV latency. One group found a temperature-induced latency in Crandell-Rees feline kidney (CRFK) cells and feline PBMC incubated with FIV at 41°C, which was reversible with return to the permissive temperature of 37°C [96]. Using methylcytosine mapping, our group found no evidence that proviral promoter CpG hypermethylation is associated with latency in peripheral CD4+ T cells or monocytes obtained from experimentally FIV-infected cats [97]. Though DNA methylation was originally implicated from *in vitro* studies of HIV latency [98,99], this association was not found in latently-infected, resting CD4+ T cells from HIV-infected individuals on ART [100], similar to our findings for FIV. We have, however, found an association between latency and a locally restrictive chromatin environment characterized by histone methylation and de-acetylation on lysine residues [91]. In the same study, we demonstrated that RNA polymerase II appeared to be paused on the latent FIV promoter, transcribing only short (between 66 and 118 bp) transcripts as mentioned above. This is especially interesting given the lack of a known *tat*-like function encoded by FIV. Histone modification (particularly acetylation) and resulting chromatin condensation is thought to be an important mechanism of latency in HIV [11,21,101-103]. A variety of pharmacologic inhibitors of histone deacetylase (HDAC) and histone methyltransferase (HMT) are able to reactivate latent FIV *ex vivo*[104], corroborating the link between FIV latency and chromatin status, and confirming that latent proviruses are capable of productive virus replication upon activation. There is substantial interest in the use of HDAC inhibitors, especially suberoylanilide hydroxamic acid (SAHA), for ALT in HIV infection [101,105-110]. Another group independently found that sodium butyrate (NaB) was able to reactive a clonal *in vitro* model of FIV latency [88]. Although not identified as such in that report, NaB is a type of HDAC inhibitor. Finally, Chan *et al.* found that the protein kinase C (PKC)-activating phorbol ester Prostratin stimulated FIV replication in a feline CD4+ T-cell line depleted of IL-2 (which was otherwise non-productive) [111]. This study suggests that PKC is important for FIV replication, and PKC-activators such as Prostratin may be useful in purging latent reservoirs. PKC activators have similarly been shown to reactivate latent HIV [112-115]. Taken together, observations regarding FIV latency reveal many similarities with the features and mechanisms of HIV-1 latency as summarized in Table 1.

**Table 1:** Summary of the features of FIV and HIV-1 latency discussed in this review

**Feature**	**FIV**	**HIV-1**
Latently infected peripheral CD4+ T cells per million, approximate	10	1-10
Primary T cell reservoir	CD4+ T cells	Central memory CD4 + T cells
Viremia in chronic infection (untreated)	Undetectable to low	Low to moderate
Accessory genes	*rev, vif, orf-A*	*rev, vif, tat, vpr, vpu, nef*
*Tat*-like function	NO	YES
Paused RNA Polymerase II detected on LTR *in vivo*	YES	YES
Detection of short, promoter-proximal transcripts *in vivo*	YES	YES
Detection of multiply-spliced viral mRNA *in vivo*	NO	NO
Promoter associated histone modifications involved in chromatin control of latency	Acetylation and methylation (others unknown)	Acetylation, methylation, phosphorylation and ubiquitination
CpG methylation of latent proviral promoter *in vivo*	NO	NO
Transcriptional reactivation by HDAC inhibitors	YES	YES
Transcriptional reactivation by HMT inhibitors	YES	YES
Transcriptional reactivation by DNMT inhibitors	Unknown	NO
Transcriptional reactivation by PKC activators	YES	YES

## Conclusions

FIV latency is a relatively new field, with a paucity of pertinent and published research on the topic, but it represents a novel and exciting model of HIV latency. FIV is known to support latent infection, both *in vitro* and *in vivo*, and many parallels have been drawn between FIV and HIV mechanisms of latency. Furthermore, many of the same drugs under investigation as potential ALT candidates for HIV have been shown to pharmacologically reactivate FIV as well. If the concept of induction therapy (reactivating latent virus to purge the reservoir) is to progress, use of an animal model of lentiviral latency will be critical to guide the research forward. Not only is latency reactivation potentially dangerous, but removing HAART from well-controlled patients may not be logistically or ethically feasible. Moreover, the dosage, timing, and sequence of ALT versus ART must be determined, and the potential for pharmacologically isolated anatomic reservoirs to reseed the latent population must be thoroughly examined. FIV may be particularly well-suited as a model of central nervous system reservoirs due to high viral loads in circulating monocytes and potential for latent microglial infection. FIV has advantages and disadvantages relative to other *in vivo* latency models as described above, but perhaps its most valuable property as a model at this early stage of ALT development is the level of natural control of the virus during the chronic phase of infection. Because viremia and cell-associated vRNA naturally progress to low or undetectable levels in peripheral lymphoid cells, the effect of reactivating agents can be extricated from ART-mediated suppression. Drug-related parameters such as efficacy, potency, and kinetics of the effect (reactivation), can therefore be more easily evaluated and “disentangled” in this animal model. In addition, since eradication strategies depend heavily on immune surveillance and effective killing of reactivated cells, it is possible (if not likely) that the compromised immune systems of infected individuals will need to be boosted in order to mount a sufficient response [116]. The extensive research into correlates of immune protection against FIV infection (including the existence of a commercial vaccine) are an additional advantage of this model [58]. In conclusion, FIV infection of the domestic cat signifies a relatively unexplored and under-recognized but potentially informative and valuable model for lentiviral latency and therapeutic reactivation in humans.

## Abbreviations

AIDS: Acquired immunodeficiency syndrome; ALT: Antilatency therapy; ART: Antiretroviral therapy; DMNT: DNA methyltransferase; FAIDS: Feline acquired immunodeficiency syndrome; FIV: Feline immunodeficiency virus; HAART: Highly active antiretroviral therapy; HIV-1: Human immunodeficiency virus-1; HDAC: Histone deacetylase; HMT: Histone methyltransferase; NaB: Sodium butyrate; PBMC: Peripheral blood mononuclear cells; PCR: Polymerase chain reaction; PKC: Protein kinase C; SIV: Simian immunodeficiency virus; SPF: Specific pathogen free.

## Competing interests

The authors declare that they have no competing interests.

## Authors’ contributions

SJM wrote the manuscript draft. BGM and EES edited all the manuscript drafts. All authors read and approved the final manuscript.
